# A novel optimization method for belief rule base expert system with activation rate

**DOI:** 10.1038/s41598-023-27498-3

**Published:** 2023-01-11

**Authors:** Gang Xiang, Jie Wang, XiaoXia Han, Shuaiwen Tang, Guanyu Hu

**Affiliations:** 1 Beihang University School of Automation Science and Electrical Engineering, Beijing, 100191 China; 2 Beijing Aerospace Automatic Control Institute, Beijing, 100854 China; 3 Guangxi Key Laboratory of Trusted Software, Guilin University of Electronic Technology, Guilin, 541004 China; 4 High-Tech Institute of Xi’an, Xi’an, 710025 China

**Keywords:** Aerospace engineering, Computer science

## Abstract

Although the belief rule base (BRB) expert system has many advantages, such as the effective use of semi-quantitative information, objective description of uncertainty, and efficient nonlinear modeling capability, it is always limited by the problem of combinatorial explosion. The main reason is that the optimization of a BRB with many rules will consume many computing resources, which makes it unable to meet the real-time requirements in some complex systems. Another reason is that the optimization process will destroy the interpretability of those parameters that belong to the inadequately activated rules given by experts. To solve these problems, a novel optimization method for BRB is proposed in this paper. Through the activation rate, the rules that have never been activated or inadequately activated are pruned during the optimization process. Furthermore, even if there is a complete data set and all rules are activated, the activation rate can also be used in the parallel optimization process of the BRB expert system, where the training data set is divided into some subprocesses. The proposed method effectively solves the combinatorial explosion problem of BRB and can make full use of quantitative data without destroying the original interpretability provided by experts. Case studies prove the advantages and effectiveness of the proposed method, which greatly expands the application fields of the BRB expert system.

## Introduction

Expert systems are one of the most traditional artificial intelligence methods and have been used in many fields, including finance, industry, medicine, and education^1^. It can express extensive knowledge and experience of a complex system and obtain the final results by the inference engine. However, in the era of big data, an expert system cannot effectively utilize multisource data from complex environments and internal systems, which limits its applications. Data-driven approaches can make up for this defect, such as neural networks^2,3^, dynamic Bayesian networks^4^, and deep learning methods^5^. However, they cannot use expert experience and domain knowledge to guide the setting of initial parameters, which brings much uncertainty and pressure to the model optimization. A semiquantitative model can combine the advantages of the above two types of models, such as the hidden Markov model (HMM)^6^ and fuzzy neural network^7^. Although the above methods have been applied in many cases, they all lack the ability to process various types of uncertainty, including randomness, fuzziness and ignorance, and lack the interpretability and credibility of the results. As an intelligent expert system and interpretable artificial intelligence method, the belief rule base (BRB) expert system^8–10^ can effectively utilize semiquantitative information, including qualitative knowledge and quantitative data, and objectively express uncertain information. Currently, increasing attention is being paid to BRBs, and many variants have been developed^11–15^.

Although BRB has many advantages, as an expert system, it will also face the problem of combinatorial explosion when the number of attributes is increased. The main reason is that the number of rules in the BRB will increase exponentially with the increase in attributes and reference levels. There are many problems to be solved for BRBs, and the most important problem is model optimization. Because of the increasing parameters in the BRB, the optimization process will expend considerable time and computing resources. For example, there are 8 attributes in distinguishing diabetes. Assuming that each attribute has 3 reference levels, the number of rules in BRB is 3^8^ = 6561. In the following sections, we will see that each rule of BRB has 12 parameters, including rule weights, attribute weights, and belief degrees of consequents. Thus, the number of parameters of the BRB is 6561 × 12 = 78,732, which means that searching for optimal parameters will run in a very high-dimensional solution space. In addition, the objective function of BRB optimization is a nonconvex, highly nonlinear, and existing equality constraint problem. Therefore, the optimization process of BRBs with a large number of parameters is very difficult to solve. On the other hand, it is also unreasonable to optimize all the parameters of the BRB because some rules may not be activated or only activated a few times by incomplete quantitative data. It is not only meaningless to optimize those rules but will also destroy the original interpretability provided by experts.

Based on the above descriptions, it is necessary to develop an effective method to solve the optimization problem of BRBs. Currently, there are 2 types of methods for this problem: (1) Dimension reduction methods, which can reduce the number of attributes, such as principal component analysis (PCA)^16^ and linear discriminant analysis (LDA)^17^. For example, Hu used PCA to reduce the characteristics of attacks in network security situation prediction by using BRB^18^. (2) Structure reduction methods, which can reduce the number of rules. For example, Zhou proposed an automatic adding and deleting criterion for belief rules in BRBs based on statistical utility^19^. Chang proposed a structure learning method for BRBs based on gray targets (GTs) and multidimensional scaling (MDS)^16^.

Although the above methods can relieve the pressure of BRB optimization to a certain extent, some shortcomings still exist. Dimension reduction methods cannot keep the original meaning of the attribute, which weakens the advantage that the BRB expert system can effectively utilize domain knowledge. Structure reduction methods are not always efficient and reach practical optimization speeds at the expense of precision. Obviously, from the view of scale reduction, the optimization problem of BRBs with a large number of parameters cannot be effectively solved. Therefore, a novel optimization method for BRBs with activation rates is proposed in this paper, where the activation rate is used to determine which rules should be optimized in a process, and then the whole optimization process of BRBs can be simplified without losing accuracy. Furthermore, in the situation that most of the rules are activated, the activation rate can also be used in the parallel process of BRB optimization, where the training data set is divided into some subprocesses. The proposed method in this paper can reduce the unnecessary optimization of those unactivated rules of the BRB expert system, which ensures that the quantitative data can be utilized as much as possible without destroying the original interpretability provided by experts.

The remainder of this paper is organized as follows. In “The basic description of the BRB expert system” section, the basic description of the BRB expert system is introduced, and the optimization problem of BRBs is analysed. In “Optimization method for BRBs” section, the novel optimization method with activation rate is proposed, and the parallelized process is constructed. In “Case studies” section, two case studies are designed to verify the effectiveness of the proposed method. The paper is concluded in “Conclusion” section.

## The basic description of the BRB expert system

### The structure of the BRB expert system

BRB is an intelligence expert system that can effectively use qualitative knowledge and quantitative data and can express most uncertainty information. The basic construction of the BRB expert system is as follows^8^.

1 $$\begin{array}{*{20}l} {R_{k} :} \hfill & {{\text{If}}\;(a_{1} \;{\text{is}}\;A_{1}^{k} ) \wedge (a_{2} \;{\text{is}}\;A_{2}^{k} ) \wedge , \ldots , \wedge (a_{M} \;{\text{is}}\;A_{M}^{k} )} \hfill \\ {} \hfill & {{\text{Then}}\{ (D_{1} ,\beta_{1,k} ),(D_{2} ,\beta_{2,k} ), \ldots ,(D_{N} ,\beta_{N,k} ),(D,\beta_{D,k} )\} } \hfill \\ {} \hfill & {{\text{with}}\;{\text{rule}}\;{\text{weight}}\;\theta_{k} \;\left( {k = 1,2, \ldots ,L} \right){\text{ and}}\;{\text{attribute}}\;{\text{weight}}\;\delta_{i} \quad \left( {i = 1,2, \ldots ,M} \right)} \hfill \\ \end{array}$$

where $R_{k}$ denotes the $kth$ rule in the belief rule base, and $a_{i} (i = 1, \ldots ,M)$ denotes the $ith$ antecedent attribute, whose referential value is $A_{i}^{k}$, $A_{i}^{k} = \left( {A_{i,1}^{k} ,A_{i,2}^{k} , \ldots A_{{i,J_{i} }}^{k} } \right)$, where $J_{i}$ denotes the number of referential levels of the $ith$ attribute. $M$ denotes the number of antecedent attributes. To simplify the problem, we assume that the number of attributes in each rule is the same. $D_{j} \left( {j = 1, \ldots ,N} \right)$ denotes the $jth$ output result, whose belief degree can be expressed by $\beta_{j,k}$. $D$ is the set that includes all the results, so the belief degree $\beta_{D,k}$ assigned to $D$ denotes the remaining belief degree. Because $\beta$ denotes the probabilities of the results, the sum of those belief degrees must equal 1, which is the constraint condition in training BRB.

The BRB expert system uses the above belief rules to construct the nonlinear relationship between the input and output of a complex system, and as a general probability, the belief degree can express various types of uncertain information in an objective world.

### The reasoning process of the BRB expert system

When data are imported into BRB, some rules are activated. The principle of the activation is that when attributes of the data match the corresponding reference levels, the transformation method is used to generate a matching degree of the attribute value relative to the reference value. The transformation method depends on the form of the attributes. If the attributes are quantitative, the matching degrees can be obtained by the equivalence transformation technique^20,21^. If the attributes are qualitative, the matching degrees can be obtained by the subjective judgment of experts^22^. All the matching degrees will construct $M$ matching degree vectors, denoted by $v_{i}$, where each vector includes $J_{i}$ matching degrees. Then a matching degree matrix $V$ can be obtained. $V$ includes an element $V_{k,i} \, \left( {k = 1, \ldots L; \, \,i = 1, \ldots M} \right)$ that is selected from $v_{i}$ according to the rules arranged by reference level. Thus the activation weight of the $kth$ rule $\omega_{k}$ can be calculated by

2 $$\omega_{k} = \frac{{\theta_{k} \prod\nolimits_{i = 1}^{M} {(V_{k,i} )^{{\overline{\delta }_{i} }} } }}{{\sum\nolimits_{k = 1}^{L} {\theta_{k} \prod\nolimits_{i = 1}^{M} {(V_{k,i} )^{{\overline{\delta }_{i} }} } } }},\,{\text{ where}}\, \, \overline{\delta }_{i} = \frac{{\delta_{i} }}{{\max \left\{ {\delta_{i} } \right\}}}$$

If the activation weight is not equal to 0, the corresponding rule is activated. Then, the following evidential reasoning (ER) rule is utilized to fuse the activated rules and finally obtain the distribution of belief degrees $\widehat{\beta }_{j}$ assigned to the results, as shown in Eq.

3 $$\begin{array}{*{20}c} {\widehat{\beta }_{j} = \frac{{\gamma \times \left[ {\prod\nolimits_{k = 1}^{L} {\left( {\omega_{k} \beta_{j,k} + 1 - \omega_{k} \sum\limits_{i = 1}^{N} {\beta_{i,k} } } \right)} - \prod\nolimits_{k = 1}^{N} {\left( {1 - \omega_{k} \sum\nolimits_{i = 1}^{N} {\beta_{i,k} } } \right)} } \right]}}{{1 - \gamma \times \left[ {\prod\nolimits_{k = 1}^{L} {\left( {1 - \omega_{k} } \right)} } \right]}}\quad } & {\left( {j = 1, \ldots ,N} \right)} \\ \end{array}$$

4 $$\gamma = \left[ {\sum\nolimits_{j = 1}^{N} {\prod\nolimits_{k = 1}^{L} {\left( {\omega_{k} \beta_{j,k} + 1 - \omega_{k} \sum\limits_{i = 1}^{N} {\beta_{i,k} } } \right) - \left( {N - 1} \right)\prod\nolimits_{k = 1}^{L} {\left( {1 - \omega_{k} \sum\limits_{i = 1}^{N} {\beta_{i,k} } } \right)} } } } \right]^{ - 1}$$

### Optimization of the BRB expert system

It can be seen from the above descriptions that BRB includes many parameters, of which the initial values are usually given by experts. These initial parameters constitute a rough BRB, which cannot produce accurate results. Therefore, parameter optimization for BRBs is necessary. The first step is to establish an optimization objective, shown as follows.

5 $$\begin{aligned} & \quad \min \left\{ {F\left( \Omega \right)} \right\} \\ & \begin{array}{*{20}l} {{\text{s}}.{\text{t}}{.}\quad 0 \le \theta_{k} \le 1,} \hfill & {k = 1, \ldots ,L} \hfill \\ {\quad \,\,\,\,\,0 \le \delta_{i} \le 1,} \hfill & {i = 1, \ldots ,M} \hfill \\ {\quad \,\,\,\,\,0 \le \beta_{j,k} \le 1,} \hfill & { \, j = 1, \ldots ,N,k = 1, \ldots ,L} \hfill \\ {\quad \,\,\,\,\,\beta_{D,k} + \sum\limits_{j = 1}^{N} {\beta_{j,k} } = 1} \hfill & {} \hfill \\ \end{array} \, \\ \end{aligned}$$

where $F\left( \Omega \right)$ denotes the objective function, which can be defined through the mean square deviation between the real values and testing results of the BRB. $\Omega$ denotes the parameters to be optimized.

Equation (3) is a highly nonlinear, highly dimensional, strongly constrained optimization problem. Therefore, the second step is to select an appropriate optimization algorithm to solve the optimization objective of the BRB^23^ used the sequential quadratic programming (SQP) algorithm to obtain the optimal parameters of BRBs^24^ proposed the projection covariance matrix adaptation evolution strategy (P-CMA-ES) algorithm, which achieves a good optimization effect.

Although the accuracy of BBR can be improved by the parameter optimization process, when the attributes and reference levels are increased, BRB has to face the problem of combinatorial explosion. As described in “Introduction” section, a large number of parameters will not only reduce the speed of optimization, which will lead to failure in the scene with high real-time requirements but also lead to a decrease in accuracy because of the difficulty of optimal solution search in high-dimensional space. The above limitation greatly restricts the application of BRBs in more complex and wide fields. Therefore, an efficient optimization method for BRBs is proposed in this paper.

## Optimization method for BRBs

A novel optimization method for BRBs with activation rates is proposed in this section. The method can be used in two different application scenarios: (1) BRBs with incomplete samples or patterns, which means that a part of the rules will never be activated or only activated a few times. By pruning these rules by using the activation rate and threshold, the optimization dimension will be greatly reduced. (2) Fully activated BRB, where all rules are activated many times. Through parallel operation by using the activation rate and threshold, optimization will be separated into many child processes, which will fundamentally solve the problem of BRB optimization. Next, the basic principles of the proposed optimization method will be introduced in two different scenarios.

### BRB optimization method with activation rate

After an in-depth analysis of the combination explosion problem, we found that when samples are incomplete or the actual system does not cover all patterns, a part of the rules will never be activated or only activated very few times in the whole training process, which is called inadequate-activated rules. However, in the traditional optimization process of BRBs, the parameters of all rules are involved in optimization, which is unreasonable. The initial parameters of the BRB are given by experts based on experience and domain knowledge. If the parameters of these nonactivated or inadequate-activated rules are optimized, then we give up expert knowledge without enough quantitative data to provide information for model learning. To solve the above problem, the activation rate for the BRB is first proposed, as follows.

6 $$ar_{k} = \frac{{an_{k} }}{{\sum\nolimits_{n = 1}^{L} {an_{n} } }}$$

where $ar_{k}$ denotes the activation rate of the $kth$ rule and $an_{k}$ denotes the number of activation times of the $kth$ rule.

To prune the nonactivated and inadequate-activated rules, a threshold $h$ of the activation rate must be given. When the activation rate is greater than the threshold $h$, the parameters of the corresponding rules can be optimized, and the remaining rules still keep the initial values given by experts.

#### Remark 1

Note that an_k_ can be obtained only after all samples are input into the initial BRB, which cannot affect the efficiency of optimization because the initial BRB is without a training process and can quickly obtain output results.

### Parallel optimization method of BRBs

The scale of the BRB can only be reduced to a certain extent through the activation rate, but when the data set is relatively complete, the scale reduction will be limited, which cannot solve the optimization problem of BRBs with a large number of parameters in essence. With the development of computer technology, parallel optimization provides a good solution, which can greatly reduce the optimization time. Theoretically, if we have enough computing units, the optimization time will surely meet the requirements of the actual system. Thus, a parallel optimization method for BRBs is proposed in this section.

Inspired by the activation rate and pruning rules, the parallelization of BRB optimization can be achieved by partitioning the data set, which can be denoted as

7 $$S = \left[ {\begin{array}{*{20}l} {s_{1,1} ,} \hfill & {\quad s_{1,2} ,} \hfill & {\quad \cdots ,} \hfill & {\quad s_{1,M} } \hfill \\ {s_{2,1} ,} \hfill & {\quad s_{2,2} ,} \hfill & {\quad \cdots ,} \hfill & {\quad s_{{{2},M}} } \hfill \\ {} \hfill & {} \hfill & {\quad \cdots } \hfill & {} \hfill \\ {s_{sn,1} ,} \hfill & {\quad s_{sn,2} ,} \hfill & {\quad \cdots ,} \hfill & {\quad s_{sn,M} } \hfill \\ \end{array} } \right]$$

where $S$ denotes the original data set, $sn$ denotes the number of samples, and $M$ denotes the number of attributes of each sample $s_{i,1} ,s_{i,2} , \cdots ,s_{i,M}$.

The parallelization steps of BRB optimization are shown as follows:

- *Step 1* First, the initial parameter values of the BRB are set according to expert knowledge.
- *Step 2* Assuming that the number of optimization subprocesses is *pn*, the training data set can be divided into *pn* parts, each of which is an average sampled from *sn* samples of the original data set.
- *Step 3* Input every sub data set into the initial BRB, and calculate the activation rate $AR_{n} = \left( {ar_{1}^{n} ,ar_{2}^{n} , \ldots ,ar_{k}^{n} } \right);\left( {n = 1,2, \ldots pn} \right)$ of each sub data set, where $AR_{i}$ denotes the activation rate set of the $ith$ sub data set. $ar_{k}^{n}$ denotes the activation rate of the $kth$ rule activated by the $nth$ sub data set.
- *Step 4* Set the threshold for the activation rate, which can decide which rules to participate in each optimization subprocess. Then, the BRB can be divided into *pn* sub-BRB models, denoted as $BRB_{n}$.
- *Step 5* The corresponding sub-BRB is assigned to different computing units and optimized independently according to the corresponding training sub data set. The optimization algorithm is P-CMA-ES, which is used to minimize the objective function shown in Eq. (3). Please refer to algorithm 1 for pseudo code of P-CMA-ES algorithm.
- *Step 6* After the above steps, we obtain *pn* groups of belief degree distributions, and each group has $sn$ belief degree distributions for the output results of $sn$ samples in the BRB. The belief degree distribution generated by the $nth$ optimization subprocess of the $ith$ testing sample can be denoted as $B_{n,i} = \left( {\hat{\beta }_{n,i}^{1} ,\hat{\beta }_{n,i}^{2} , \ldots ,\hat{\beta }_{n,i}^{N} } \right)$. To obtain the final belief degree distribution, the weighted average method is utilized, and the weight of the $nth$ distribution can be determined by $pw_{n}$

8 $$p\omega_{n} = \frac{{\sum\nolimits_{k = 1}^{L} {an_{k}^{n} } }}{{\sum\nolimits_{n = 1}^{pn} {\sum\nolimits_{k = 1}^{L} {an_{k}^{n} } } }} \times \left( {pn - 1} \right)$$

where $an_{k}^{n}$ denotes the number of activation times of the $kth$ rule in $BRB_{n}$. Then, the final belief degrees distribution of $ith$ sample $B_{f}^{i}$ can be obtained by Eq. (8), the final results of the BRB can be obtained by Eq. (9).

9 $$B_{f}^{i} { = }\left( {\frac{{\sum\nolimits_{n = 1}^{pn} {p\omega_{n} \times \hat{\beta }_{n,i}^{1} } }}{pn},\frac{{\sum\nolimits_{n = 1}^{pn} {p\omega_{n} \times \hat{\beta }_{n,i}^{2} } }}{pn}, \ldots ,\frac{{\sum\nolimits_{n = 1}^{pn} {p\omega_{n} \times \hat{\beta }_{n,i}^{N} } }}{pn}} \right)$$

10 $${\rm Z}_{i} = \sum\limits_{j = 1}^{N} {B_{f,j}^{i} \times D_{j} ;} \quad \left( {i = 1, \ldots ,sn} \right)$$

#### Remark 2

The above optimization subprocesses are independent of each other. The weighted average operation for the final belief degree distribution is executed only when the optimization is completed.


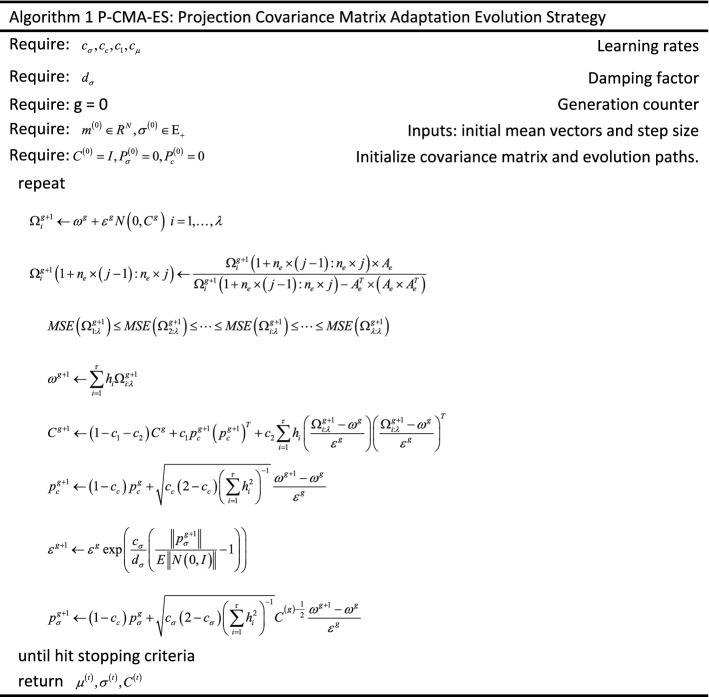


## Case studies

To verify the superiority of the proposed method, two cases, “Health status assessment of laser gyro” and “Leak size estimation of oil pipeline”, were used for verification.

### Health status assessment of laser gyro

#### Problem formulation

A laser gyro is a precision instrument in the navigation control system. Its state parameters are zero-order drift coefficient, first-order drift coefficient, X-axis gyroscope light intensity voltage. When these parameters exceed the calibration threshold, it means that the laser gyro has failed, and the navigation control system will fail at this time. However, when these parameters are within the threshold, the laser gyro will also show different states. At this time, evaluating their health is also a necessary means to measure whether the laser gyro meets the navigation accuracy. Therefore, this case studies the health assessment of laser gyro, using the following data sets.

**Figure 1:**
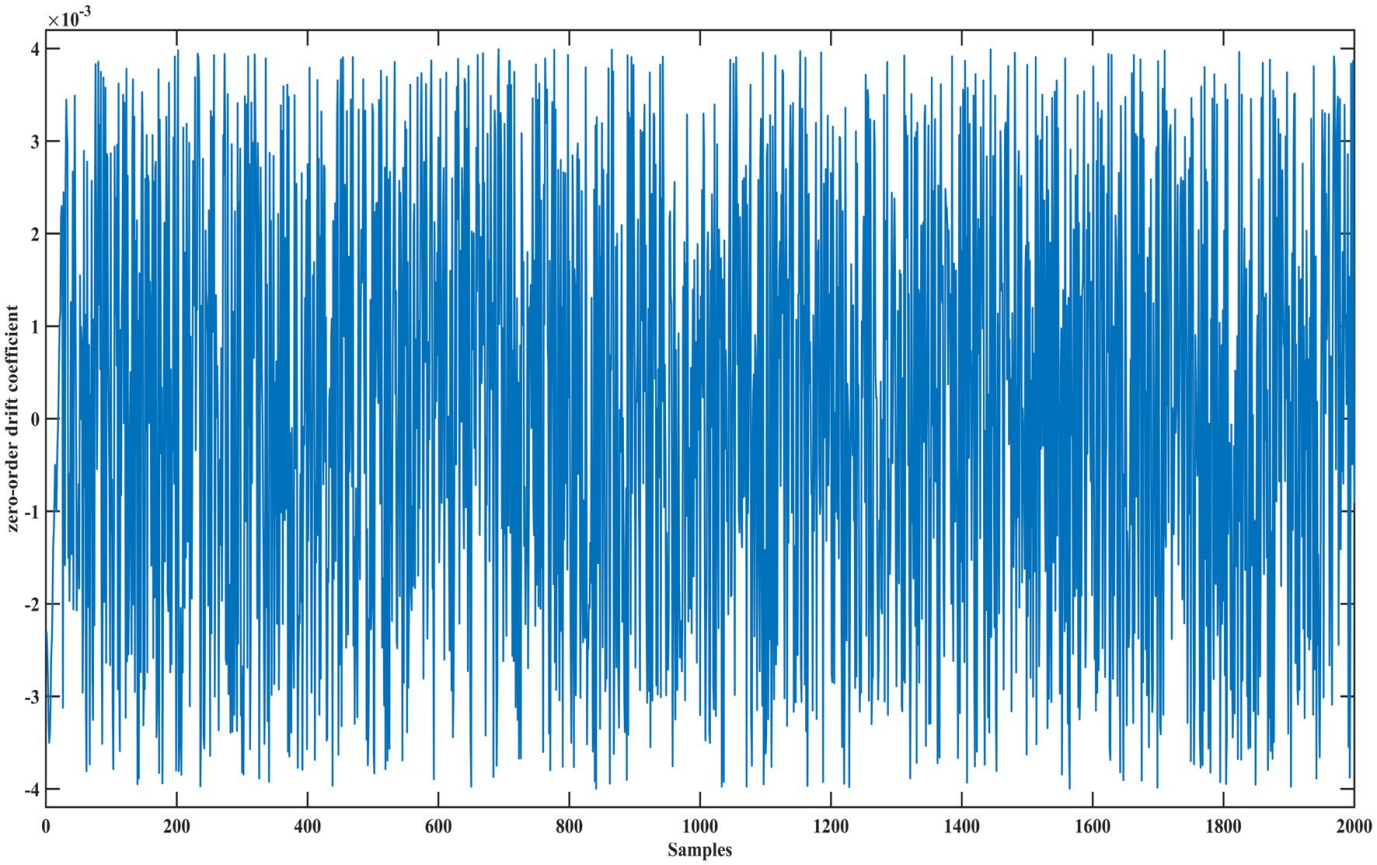
The zero-order drift coefficient of the laser gyro.

**Figure 2:**
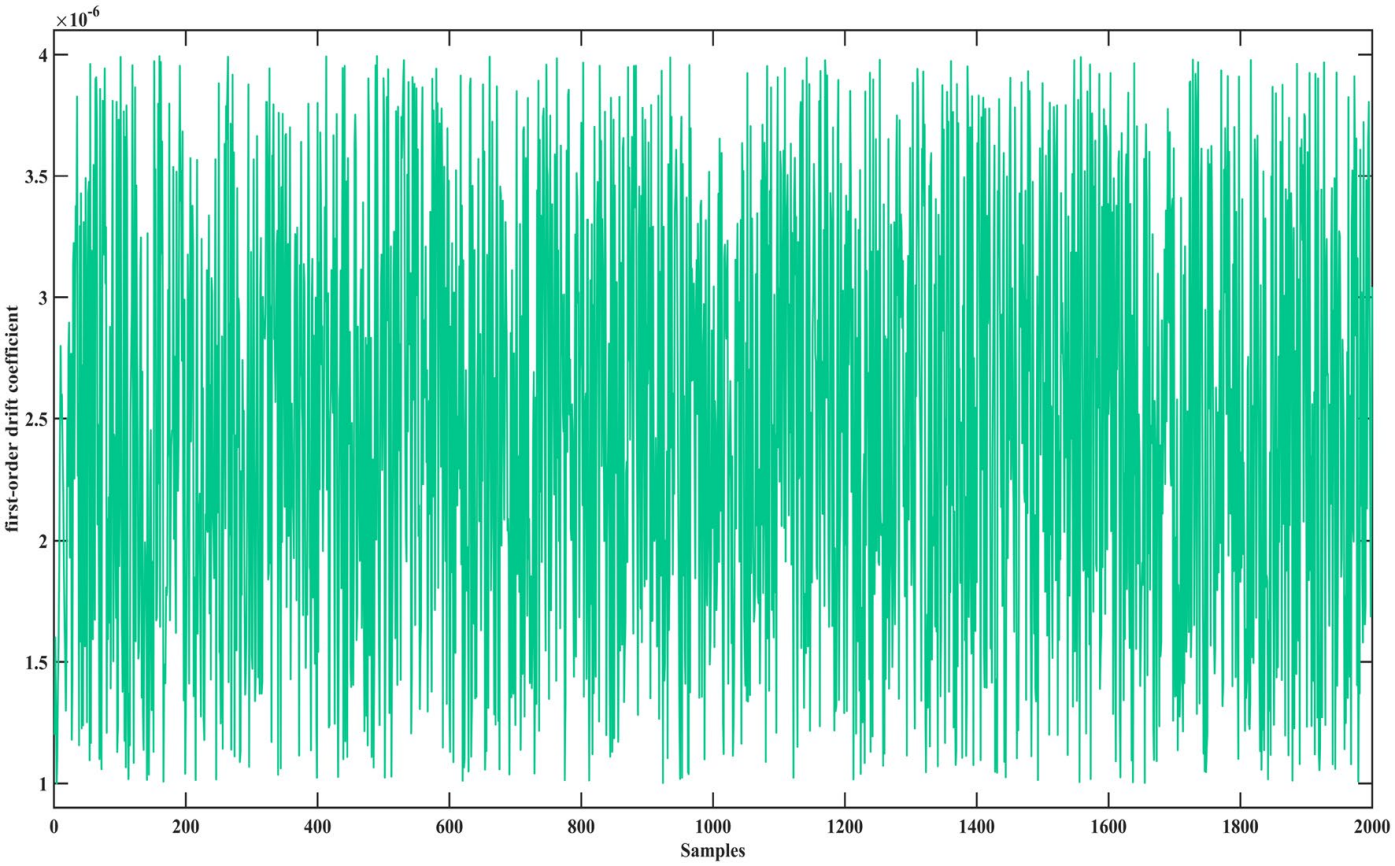
The first-order drift coefficient of the laser gyro.

**Figure 3:**
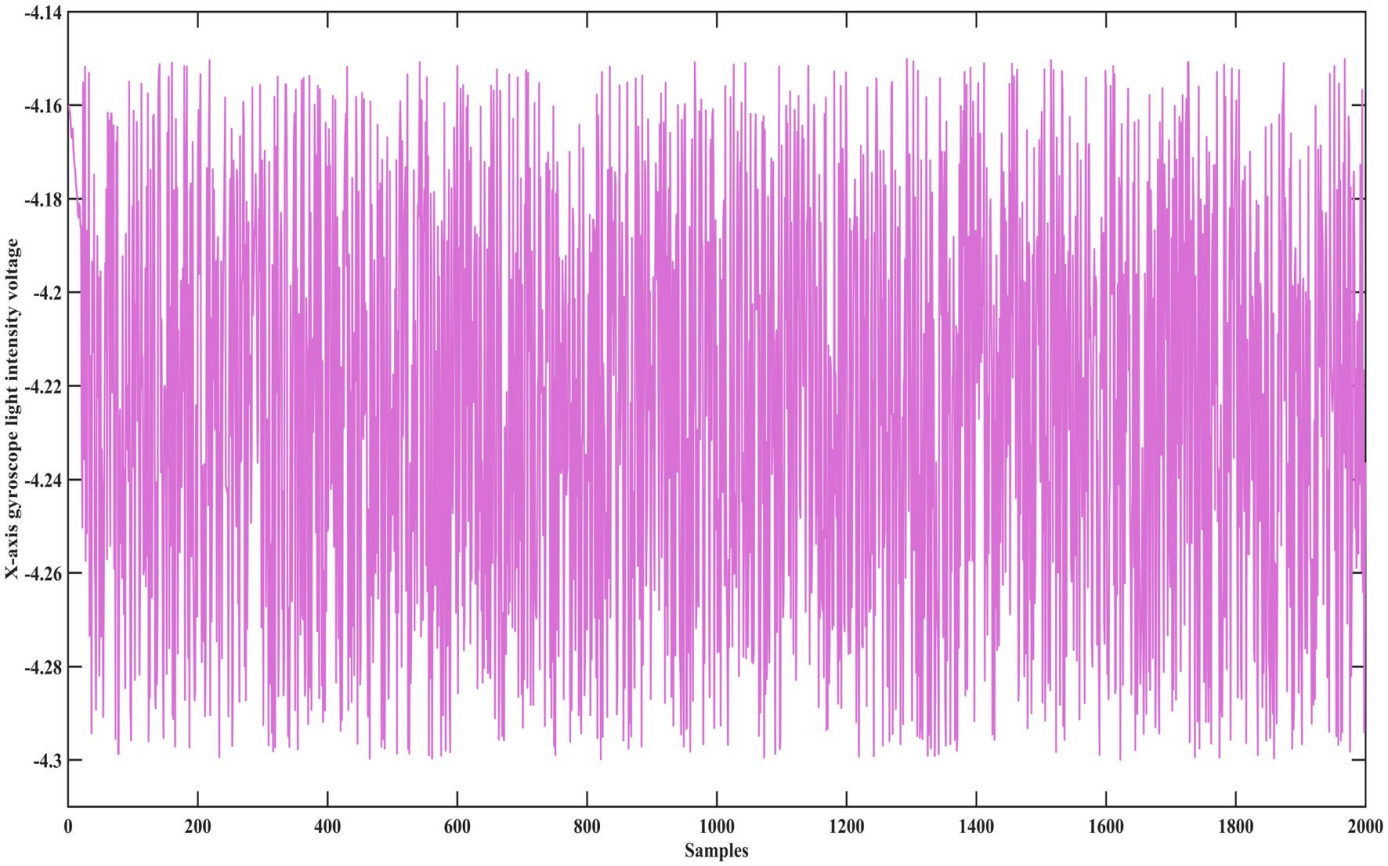
The X-axis gyroscope light intensity voltage of the laser gyro.

**Figure 4:**
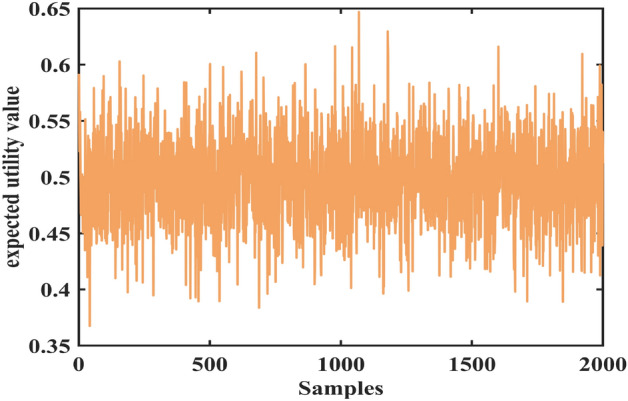
The expected utility value of the laser gyro.

In this case, the data set of the laser gyro is used to prove the advantages of the proposed method. This data set contains a zero-order drift coefficient, first-order drift coefficient, X-axis gyroscope light intensity voltage, and expected utility value. The data set has 2000 samples, as shown in Figs. 1, 2, 3 and 4.

First, we can establish a BRB expert system according to expert experience or domain knowledge. The reference values of the zero-term drift coefficient, first-term drift coefficient, and X-axis gyroscope light intensity voltage are shown in Tables 1, 2 and 3. Thus the BRB expert system of laser gyroscope health status detection can be described

11 $$\begin{array}{*{20}l} {R_{k} :} \hfill & {IF \, \,\left( {zero - term\, \, drift \, \,coefficient\,{\text{ is}}\, \, A_{1}^{k} } \right) \wedge \left( {first - term \, \,drift \, \,coefficient \, \,{\text{is }}A_{2}^{k} } \right) \wedge \left( {X - axis\,{\text{ is }}\,A_{3}^{k} } \right)} \hfill \\ {} \hfill & {Then\, \, laser\, \, gyroscope\, \, health\, \, status \, \,is \, \,\left\{ {\left( {H, \, \beta_{1}^{k} } \right), \, \left( {SH, \, \beta_{2}^{k} } \right), \, \left( {UH, \, \beta_{3}^{k} } \right)} \right\}} \hfill \\ {} \hfill & {with \, \,rule\, \, weight\, \, \theta_{k} \, \,and\, \, attribute \, \,weight\, \, \delta_{i} } \hfill \\ \end{array}$$

where $H,SH,UH$ denote the reference values of the laser gyroscope health status, as shown in Table 4. The other parameters of the BRB expert system are shown in Table 5.

**Table 1:** The reference values and points of the zero-order term drift coefficient.

Linguistic terms	$NL$	$NS$	$PS$	$PL$
Values	$- 4 \times 10^{ - 3}$	$- 1.3 \times 10^{ - 3}$	$1.3 \times 10^{ - 3}$	$4 \times 10^{ - 3}$

**Table 2:** The reference values and points of the primary term drift coefficient.

Linguistic terms	$PS$	$PM$	$PL$	$PVL$
Values	$1 \times 10^{ - 6}$	$2 \times 10^{ - 6}$	$3 \times 10^{ - 6}$	$4 \times 10^{ - 6}$

**Table 3:** The reference values and points of the X-axis gyroscope light intensity voltage.

Linguistic terms	$NL$	$NM$	$NS$	$NVS$
Values	$- 4.3$	$- 4.25$	$- 4.2$	$- 4.15$

**Table 4:** The reference values and points of laser gyroscope health status.

Linguistic terms	$H$	$SH$	$UH$
Values	$0$	$2$	$4$

**Table 5:** Initial parameters of the BRB expert system.

Rule	Weight	Attributes			Belief degrees distribution
		$a_{1} \left( t \right)$	$a_{2} \left( t \right)$	$a_{2} \left( t \right)$	$\left\{ {H,SH,UH} \right\}$
1	1	NL	PS	NL	$\left\{ {1,0,0} \right\}$
2	1	NL	PS	NM	$\left\{ {0.9,0.1,0} \right\}$
3	1	NL	PS	NS	$\left\{ {0.8,0.2,0} \right\}$
4	1	NL	PS	NVS	$\left\{ {{0}{\text{.7,0}}{.3,0}} \right\}$
5	1	NL	PM	NL	$\left\{ {{0}{\text{.9,0}}{.1,0}} \right\}$
6	1	NL	PM	NM	$\left\{ {{0}{\text{.8,0}}{.1,0}{\text{.1}}} \right\}$
7	1	NL	PM	NS	$\left\{ {{0}{\text{.7,0}}{.2,0}{\text{.1}}} \right\}$
8	1	NL	PM	NVS	$\left\{ {{0}{\text{.7,0}}{.3,0}} \right\}$
9	1	NL	PL	NL	$\left\{ {{0}{\text{.8,0}}{.2,0}} \right\}$
10	1	NL	PL	NM	$\left\{ {{0}{\text{.7,0}}{.2,0}{\text{.1}}} \right\}$
11	1	NL	PL	NS	$\left\{ {{0}{\text{.6,0}}{.3,0}{\text{.1}}} \right\}$
12	1	NL	PL	NVS	$\left\{ {{0}{\text{.6,0}}{.4,0}} \right\}$
13	1	NL	PVL	NL	$\left\{ {{0}{\text{.8,0}}{.2,0}} \right\}$
14	1	NL	PVL	NM	$\left\{ {{0}{\text{.7,0}}{.3,0}} \right\}$
15	1	NL	PVL	NS	$\left\{ {{0}{\text{.6,0}}{.4,0}} \right\}$
16	1	NL	PVL	NVS	$\left\{ {{0}{\text{.5,0}}{.4,0}{\text{.1}}} \right\}$
17	1	NS	PS	NL	$\left\{ {{0}{\text{.8,0}}{.2,0}} \right\}$
18	1	NS	PS	NM	$\left\{ {{0}{\text{.7,0}}{.2,0}{\text{.1}}} \right\}$
19	1	NS	PS	NS	$\left\{ {{0}{\text{.6,0}}{.3,0}{\text{.1}}} \right\}$
20	1	NS	PS	NVS	$\left\{ {{0}{\text{.5,0}}{.4,0}{\text{.1}}} \right\}$
21	1	NS	PM	NL	$\left\{ {{0}{\text{.5,0}}{.3,0}{\text{.2}}} \right\}$
22	1	NS	PM	NM	$\left\{ {{0}{\text{.4,0}}{.5,0}{\text{.1}}} \right\}$
23	1	NS	PM	NS	$\left\{ {{0}{\text{.3,0}}{.7,0}} \right\}$
24	1	NS	PM	NVS	$\left\{ {{0}{\text{.3,0}}{.6,0}{\text{.1}}} \right\}$
25	1	NS	PL	NL	$\left\{ {{0}{\text{.1,0}}{.6,0}{\text{.3}}} \right\}$
26	1	NS	PL	NM	$\left\{ {{0}{\text{.1,0}}{.6,0}{\text{.3}}} \right\}$
27	1	NS	PL	NS	$\left\{ {{0}{\text{.1,0}}{.6,0}{\text{.3}}} \right\}$
28	1	NS	PL	NVS	$\left\{ {{0}{\text{.1,0}}{.6,0}{\text{.3}}} \right\}$
29	1	NS	PVL	NL	$\left\{ {{0}{\text{.1,0}}{.3,0}{\text{.6}}} \right\}$
30	1	NS	PVL	NM	$\left\{ {{0}{\text{.1,0}}{.5,0}{\text{.4}}} \right\}$
31	1	NS	PVL	NS	$\left\{ {{0}{\text{.1,0}}{.3,0}{\text{.6}}} \right\}$
32	1	NS	PVL	NVS	$\left\{ {{0}{\text{.1,0}}{.2,0}{\text{.7}}} \right\}$
33	1	PS	PS	NL	$\left\{ {{0}{\text{.3,0}}{.5,0}{\text{.2}}} \right\}$
34	1	PS	PS	NM	$\left\{ {{0}{\text{.1,0}}{.6,0}{\text{.3}}} \right\}$
35	1	PS	PS	NS	$\left\{ {{0}{\text{.1,0}}{.5,0}{\text{.4}}} \right\}$
36	1	PS	PS	NVS	$\left\{ {{0}{\text{.1,0}}{.4,0}{\text{.5}}} \right\}$
37	1	PS	PM	NL	$\left\{ {{0}{\text{.1,0}}{.6,0}{\text{.3}}} \right\}$
38	1	PS	PM	NM	$\left\{ {{0}{\text{.1,0}}{.7,0}{\text{.2}}} \right\}$
39	1	PS	PM	NS	$\left\{ {{0,0}{\text{.6,0}}{.4}} \right\}$
40	1	PS	PM	NVS	$\left\{ {{0,0}{\text{.5,0}}{.5}} \right\}$
41	1	PS	PL	NL	$\left\{ {{0,0}{\text{.5,0}}{.5}} \right\}$
42	1	PS	PL	NM	$\left\{ {{0,0}{\text{.6,0}}{.4}} \right\}$
43	1	PS	PL	NS	$\left\{ {{0,0}{\text{.5,0}}{.5}} \right\}$
44	1	PS	PL	NVS	$\left\{ {{0,0}{\text{.4,0}}{.6}} \right\}$
45	4	PS	PVL	NL	$\left\{ {{0}{\text{.1,0}}{.4,0}{\text{.5}}} \right\}$
46	1	PS	PVL	NM	$\left\{ {{0,0}{\text{.4,0}}{.6}} \right\}$
47	1	PS	PVL	NS	$\left\{ {{0,0}{\text{.5,0}}{.5}} \right\}$
48	1	PS	PVL	NVS	$\left\{ {{0,0}{\text{.4,0}}{.6}} \right\}$
49	1	PL	PS	NL	$\left\{ {{0}{\text{.2,0}}{.3,0}{\text{.5}}} \right\}$
50	1	PL	PS	NM	$\left\{ {{0}{\text{.3,0}}{.2,0}{\text{.5}}} \right\}$
51	1	PL	PS	NS	$\left\{ {{0}{\text{.1,0}}{.4,0}{\text{.5}}} \right\}$
52	1	PL	PS	NVS	$\left\{ {{0,0}{\text{.5,0}}{.5}} \right\}$
53	1	PL	PM	NL	$\left\{ {{0}{\text{.3,0}}{.2,0}{\text{.5}}} \right\}$
54	1	PL	PM	NM	$\left\{ {{0}{\text{.2,0}}{.3,0}{\text{.5}}} \right\}$
55	1	PL	PM	NS	$\left\{ {{0}{\text{.1,0}}{.4,0}{\text{.5}}} \right\}$
56	1	PL	PM	NVS	$\left\{ {{0,0}{\text{.4,0}}{.6}} \right\}$
57	1	PL	PL	NL	$\left\{ {{0}{\text{.1,0}}{.4,0}{\text{.5}}} \right\}$
58	1	PL	PL	NM	$\left\{ {{0}{\text{.1,0}}{.4,0}{\text{.5}}} \right\}$
59	1	PL	PL	NS	$\left\{ {{0}{\text{.1,0}}{.3,0}{\text{.6}}} \right\}$
60	1	PL	PL	NVS	$\left\{ {{0,0}{\text{.3,0}}{.7}} \right\}$
61	1	PL	PVL	NL	$\left\{ {{0}{\text{.1,0}}{.1,0}{\text{.8}}} \right\}$
62	1	PL	PVL	NM	$\left\{ {{0,0}{\text{.2,0}}{.8}} \right\}$
63	1	PL	PVL	NS	$\left\{ {{0,0}{\text{.1,0}}{.9}} \right\}$
64	1	PL	PVL	NVS	$\left\{ {0,0,1} \right\}$

As described in the above sections, the data set may not cover all patterns about the zero-order drift coefficient, first-order drift coefficient and X-axis gyroscope light intensity voltage, which means that perhaps only a part of the rules shown in Table 6 can be activated. To prove this point, the laser gyro data samples are input into the above BRB expert system. The samples are divided into a training data set and a testing data set. The training data set includes 500 samples, which are regularly selected from the 2000 samples, and the remaining 1500 samples are used as a testing data set. The P-CMA-ES algorithm is utilized as an optimization tool of the BRB expert system, and to ensure the fairness of the optimization process, the parameters and iterations are the same for different conditions.

**Table 6:**
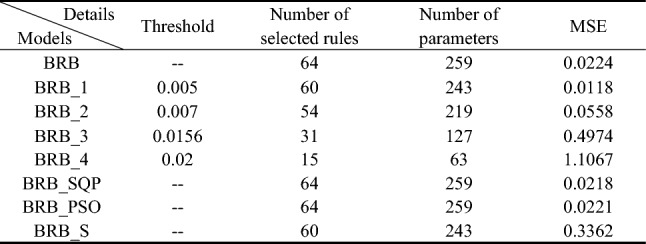
The details and final testing results generated by different BRBs.

#### Case 1-optimization process of a BRB using activation rates

**Figure 5:**
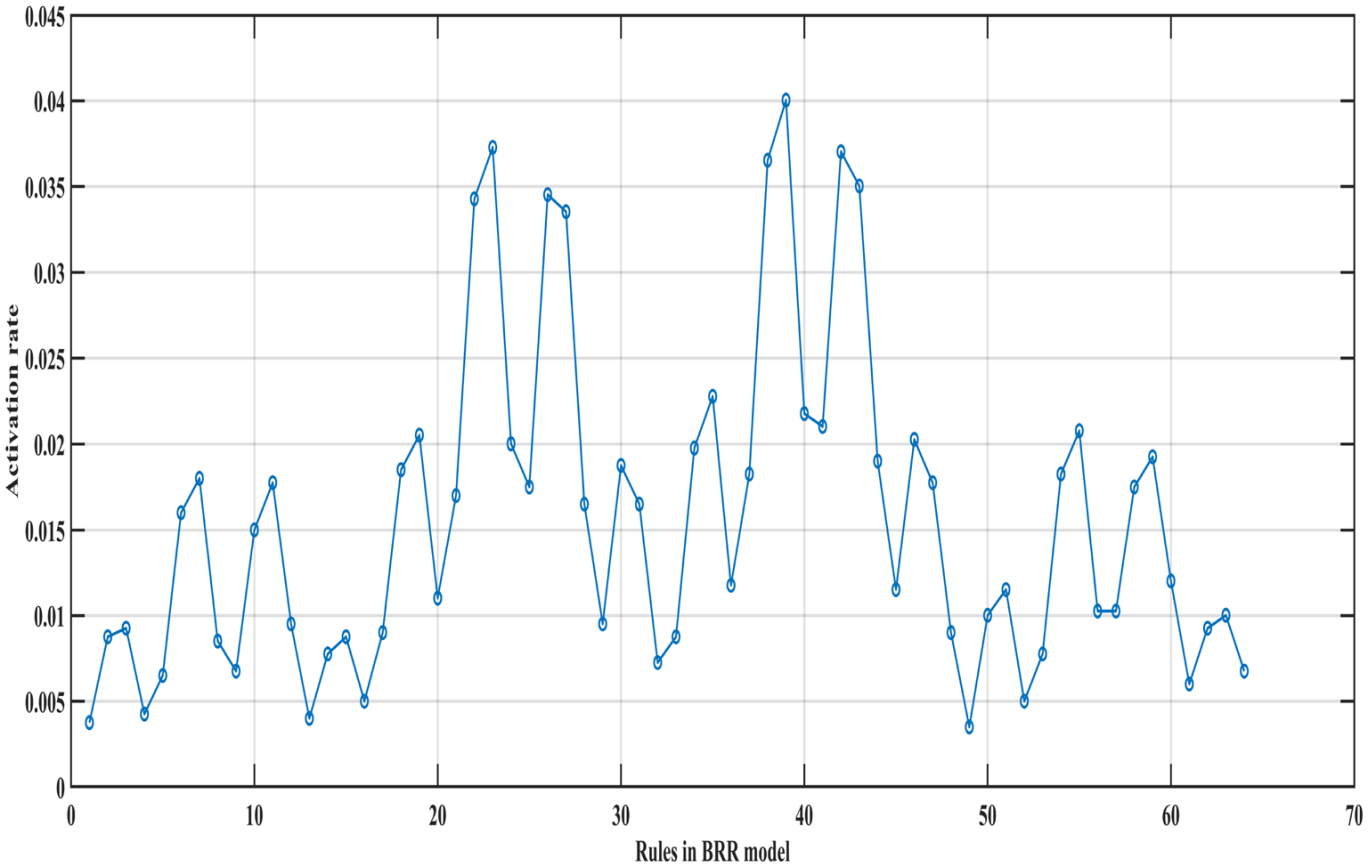
The activation rates of 64 rules in the BRB expert system.

Figure 5 shows the activation rates of 64 rules in the BRB expert system when the training data set is entered. It can be seen that the activation rates of different rules are not the same, and the activation rates of some rules are even very small and will hardly be activated.

Four BRBs with different activation rate thresholds are established for comparison with the traditional BRB. In these BRBs, the rule whose activation rate is larger than the threshold value will be selected to participate in the optimization process. Thus, the different threshold values will retain different numbers of rules. The optimization algorithm is P-CMA-ES, and the iteration time is 100. Table 6 shows the details and testing results generated by different BRBs. Where BRB stands for traditional BRB, BRB_1 to BRB_4 represents four BRBs with different activation thresholds, BRB_SQP stands for BRB using Sequential Quadratic Programming optimization algorithm, BRB_PSO stands for BRB using Particle Swarm Optimization algorithm, BRB_S stands for BRB using only the selected rules. Table 7 shows the health status distribution of different laser gyros under different BRBs.

**Table 7:**
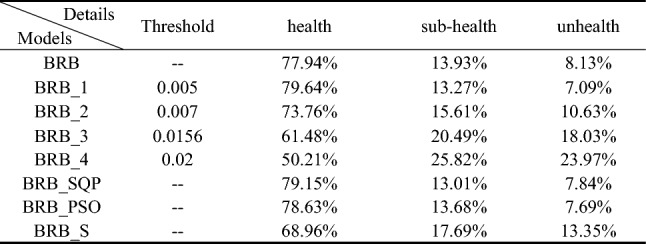
Average belief degrees distribution of different BRBs.

It can be seen that BRB_1 with fewer rules generates the best result. The mean square error (MSE) of BRB_1 with 243 optimization parameters is even smaller than that of the traditional BRB with 259 optimization parameters.

**Figure 6:**
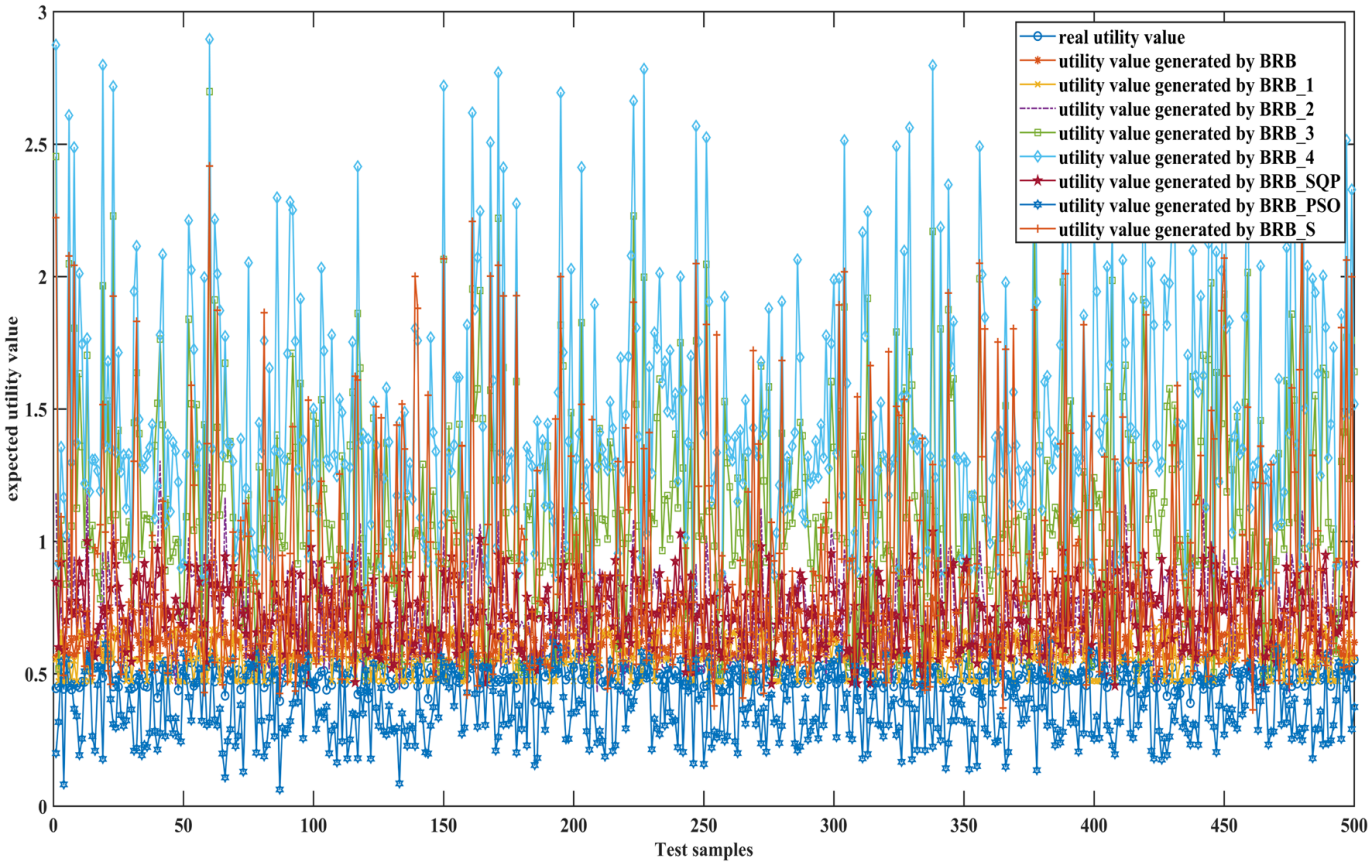
The 1–500 comparative results generated by different BRBs.

**Figure 7:**
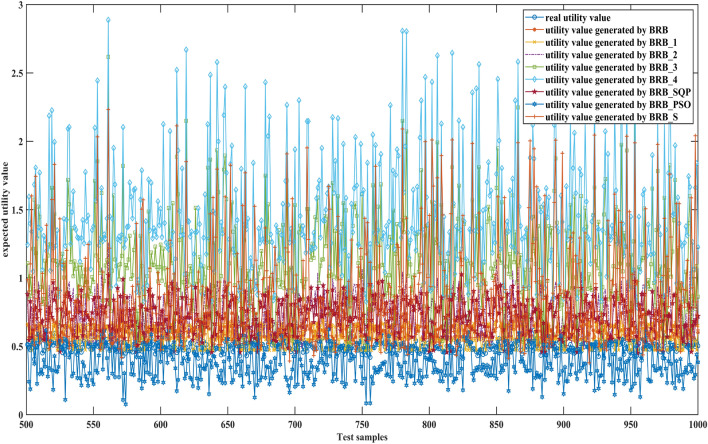
The 501–1000 comparative results generated by different BRBs.

**Figure 8:**
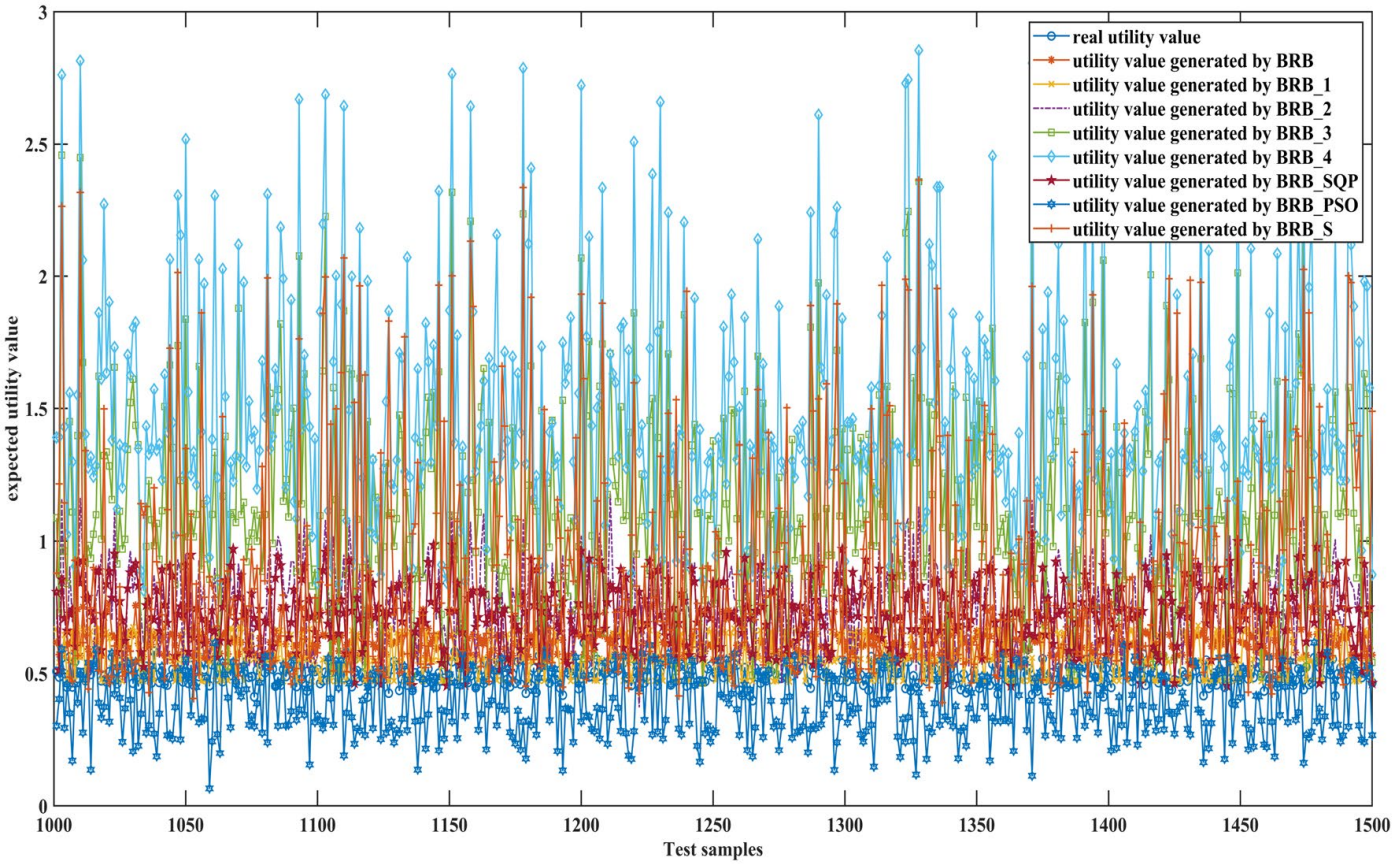
The 1001–1500 comparative results generated by different BRBs.

Figures 6, 7 and 8 can better demonstrate the results of comparative experiments, where 1500 samples are divided into three parts.

##### Remark 3

Note that the number of selected rules shown in Table 6 does not mean that the BRB expert system only has these rules; the remaining rules are also included. They do not participate in the optimization process.

##### Remark 4

As seen from the above experimental results, although BRB_1 achieves the best accuracy with minimum rules in the optimization process, the accuracy is not sufficient because of the few iterations of the P-CMA-ES algorithm. It is obvious that, in theory, further optimization will not have an obvious effect.

#### Case 2-parallel processing for BRBs using activation rates

**Figure 9:**
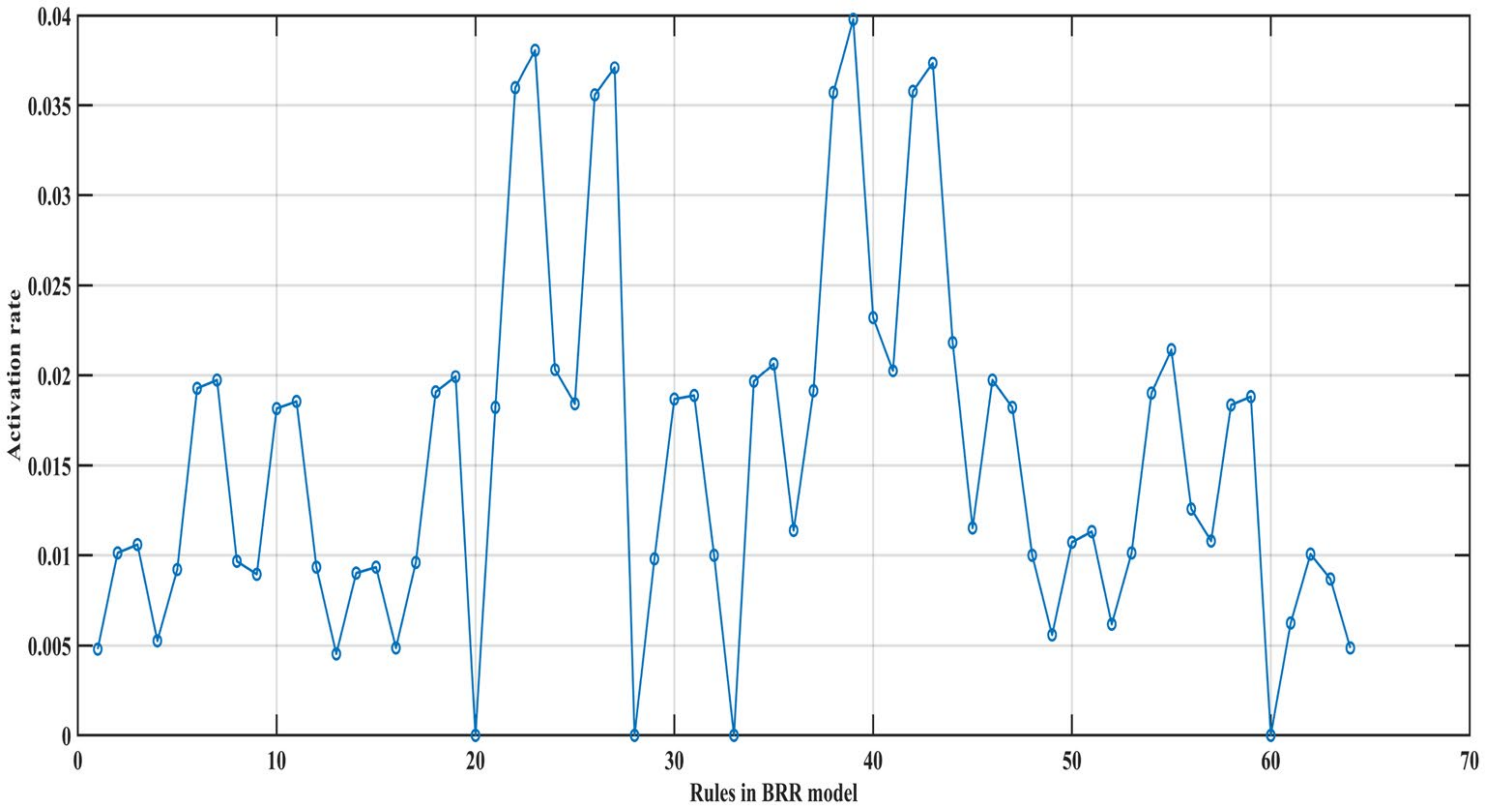
The activation rates of 64 rules generated by all samples.

In this section, all samples are treated as training data to activate more rules in the BRB to start up the parallel optimization process of the BRB. Figure 9 shows the activation rates generated by training data. The activation rates of 64 rules are more balanced than the activation rates shown in Fig. 5 of case 4.1.2. In Fig. 9, most rules have been activated, although some rules are activated less frequently. When more rules participate in the optimization process, the number of parameters to be optimized will increase dramatically. Therefore, the method of decomposing the data set is proposed to generate independent optimization subprocesses.

##### Remark 5

Note that the training data set of optimization subprocesses is divided into some independent parts, and each part is average sampled from all samples in the original data set. The number of parts of the training data set is equal to the number of optimization subprocesses whose parameters are the same as those in case 4.1.2.

**Figure 10:**
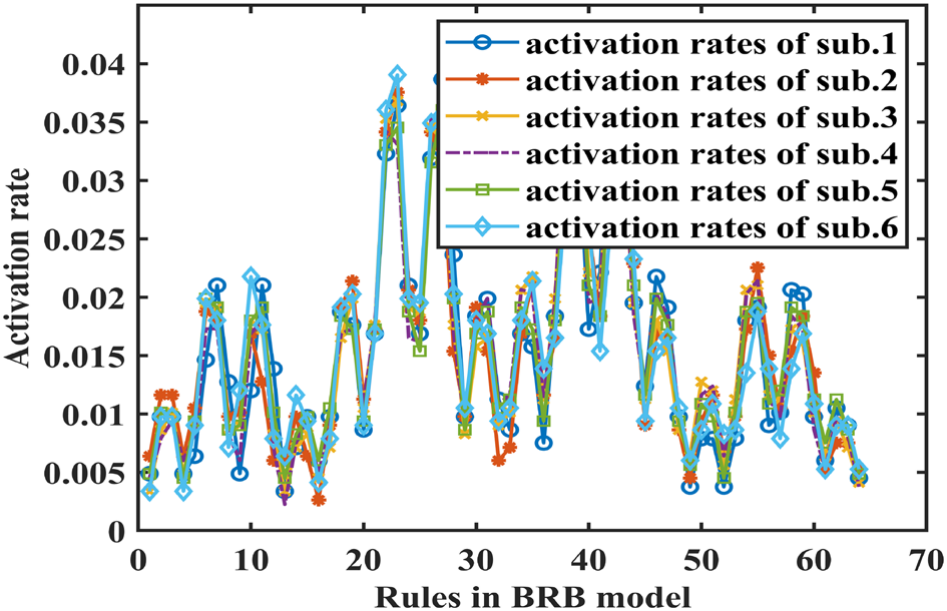
The activation rates of 64 rules generated by sub.1–6.

By using the parallel optimization method described in “Parallel optimization method of BRBs” section, the training data sets are divided into 6 parts, each of which belongs to the corresponding 6 sub-BBR models, denoted as sub.1–6. Thus, the parallel optimization processes can also be divided into 6 parts. The operating environment uses the MATLAB parallel toolkit with an Intel Core i7-8750H 2.2 GHz CPU and 16 GB memory. The optimization algorithm is P-CMA-ES, and the iteration time is 100. The activation rates of 64 rules generated by subs.1–6 are shown in Fig. 10.

In this case, the activation rate threshold of the parallel optimization is 0.001; then, the number of rules involved in the optimization of each sub-BRB are shown in Table 8.

**Table 8:** The number of rules involved in optimization in each sub-BRB.

Sub-BRBs	Sub1	Sub2	Sub3	Sub4	Sub5	Sub6
The number of rules	38	42	43	43	44	41

Thus, the 6 sub-BRBs are assigned to 6 optimization subprocesses and optimized independently. The comparison results between the original BRB without using the activation rate and optimization parallelization, the BRB with the activation rate but without optimization parallelization (nonparallelization BRB_a), and the BRB with the activation rate and optimization parallelization (parallelization BRB_a) are shown in Table 9.

**Table 9:**
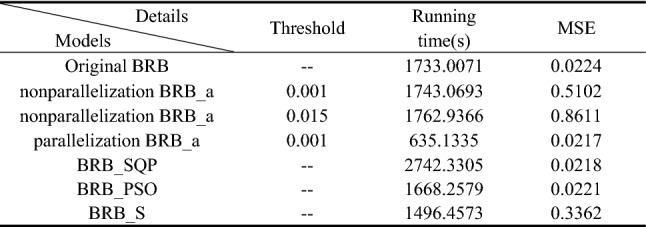
The comparison results between different BRBs.

In Table 9, parallelization BRB_a uses the least time to obtain the best results. Its running time is approximately one-third that of other models. It can be predicted that with the increase in the number of processors, the running time will decrease without affecting the accuracy.

The experimental results show that the BRB expert system with activation rate has higher accuracy than the initial BRB or other BRB optimization algorithms in the process of laser gyro health state assessment, and the time spent is also very little. It effectively solves the problem of BRB expert system combination explosion.

### Leak size estimation of oil pipeline

#### Problem formulation

**Figure 11:**
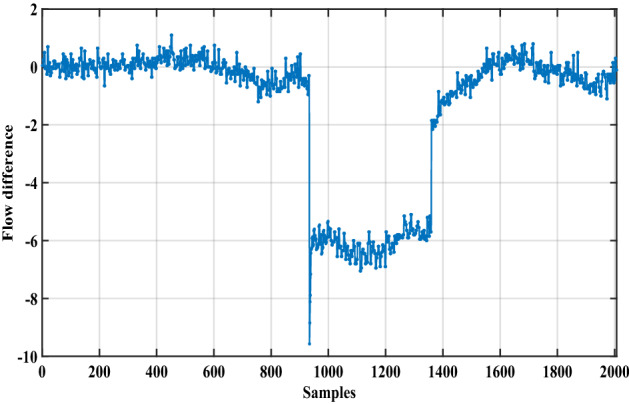
The flow difference of the oil pipeline.

**Figure 12:**
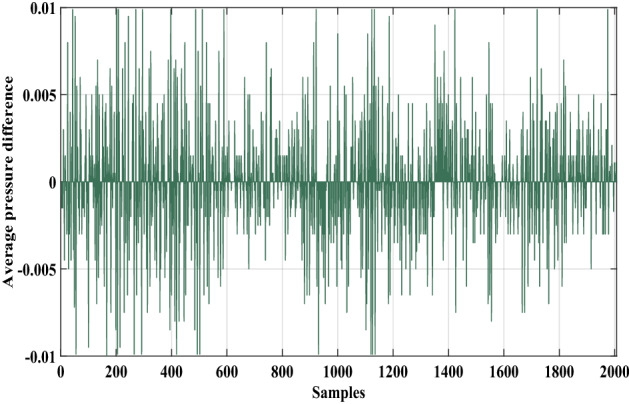
The average pressure difference of the oil pipeline.

**Figure 13:**
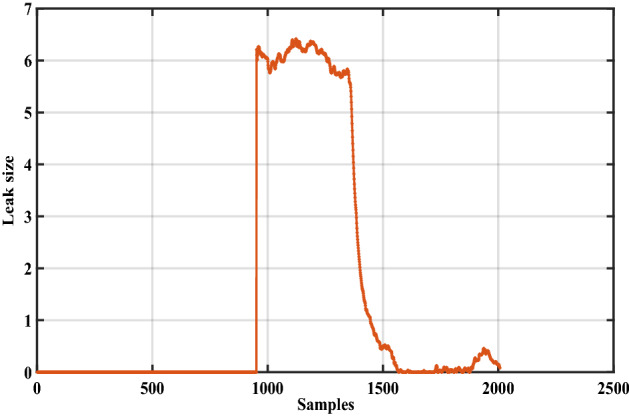
The leak size of the oil pipeline.

In this case, an actual data set of an oil pipeline leak in Britain is used to prove the advantage of the proposed method^1^. This oil pipeline is a hundred kilometers long, and when the pipeline leaks, the flow rate and pressure of the oil in the pipeline will change according to a certain mode. The data set has 2007 samples which include flow difference, average pressure difference, and leak size, as shown in Figs. 11, 12 and 13.

The reference values of flow difference and pressure difference are shown in Tables 10, 11. Thus, the BRB expert system of pipeline leak detection can be described as

$$\begin{array}{*{20}l} {R_{k} :} \hfill & {If\, \, flow\_difference \, \,is\, \, A_{1}^{k} \, \wedge \,pressure\_difference\, \, is\, \, A_{2}^{k} } \hfill \\ {} \hfill & {Then \, \,Leak\_size\, \, is\, \, \left\{ {\left( {Z,\beta_{1}^{k} } \right),\left( {VS,\beta_{2}^{k} } \right),\left( {M,\beta_{3}^{k} } \right),\left( {H,\beta_{4}^{k} } \right),\left( {VH,\beta_{5}^{k} } \right)} \right\}} \hfill \\ {} \hfill & {with \, \,rule\, \, weight\, \, \theta_{k} \, \,and\, \, attribute\, \, weight\, \, \delta_{i} } \hfill \\ \end{array} \,$$

where *Z*, *VS*, *M*, *H*, *VH* denote the reference values of leak size, as shown in Table 12. The other parameters of the BRB expert system are shown in Table 13.

**Table 10:** Referential values of flow difference.

Linguistic terms	Negative large	Negative medium	${\text{Negative}}\,{\text{ small}}$	${\text{Negative }}\,{\text{very}}\,{\text{ small}}$
Values	− 10	− 5	− 3	− 1

Linguistic terms	${\text{Zero}}$	${\text{Positive}}\,{\text{ small}}$	${\text{Positive }}\,{\text{medium}}$	${\text{Positive }}\,{\text{large}}$
Values	0	1	2	3

**Table 11:** Referential values of average pressure difference.

Linguistic terms	${\text{Negative}}\,{\text{ large}}$	${\text{Negative}}\,{\text{ medium}}$	${\text{Negative}}\,{\text{ small}}$	${\text{Negative}}\,{\text{ very}}\,{\text{ small}}$
Values	− 0.042	− 0.025	− 0.01	void

Linguistic terms	Zero	${\text{Positive}}\,{\text{ small}}$	${\text{Positive}}\,{\text{ medium}}$	${\text{Positive}}\,{\text{ large}}$
Values	0	0.01	0.025	0.042

**Table 12:** Referential values of leak size.

Linguistic terms	Zero	${\text{Very}}\,{\text{ small}}$	${\text{Medium}}$	${\text{High}}$	${\text{Very}}\,{\text{ high}}$
Values	0	2	4	6	8

**Table 13:** Initial parameters of BRB expert system.

Rule	Weight	Flow and pressure difference with weight = 1	Belief degrees distribution for $\left\{ {Z, \, VS, \, M,H \, ,VH} \right\}$
1	1	Negative large, Negative large	0 0 0 0 1
2	1	Negative large, Negative medium	0 0 0 0.3 0.7
3	1	Negative large, Negative small	0 0 0.2 0.8 0
4	1	Negative large, Zero	0 0 0.8 0.2 0
5	1	Negative large, Positive small	0.65 0.35 0 0 0
6	1	Negative large, Positive medium	0.85 0.15 0 0 0
7	1	Negative large, Positive large	0.95 0.05 0 0 0
8	1	Negative medium, Negative large	0 0 0.1 0.9 0
9	1	Negative medium, Negative medium	0 0 0.7 0.3 0
10	1	Negative medium, Negative small	0 0.7 0.3 0 0
11	1	Negative medium, Zero	0 0.9 0.1 0 0
12	1	Negative medium, Positive small	0.8 0.2 0 0 0
13	1	Negative medium, Positive medium	0.9 0.1 0 0 0
14	1	Negative medium, Positive large	0.99 0.01 0 0 0
15	1	Negative small, Negative large	0 0 0.4 0.6 0
16	1	Negative small, Negative medium	0 0 0.8 0.2 0
17	1	Negative small, Negative small	0 0.3 0.6 0.1 0
18	1	Negative small, Zero	0.1 0.7 0.2 0 0
19	1	Negative small, Positive small	0.7 0.3 0 0 0
20	1	Negative small, Positive medium	0.9 0.1 0 0 0
21	1	Negative small, Positive large	1 0 0 0 0
22	1	Negative very small, Negative large	0.02 0.11 0.39 0.48 0
23	1	Negative very small, Negative medium	0.1 0.78 0.12 0 0
24	1	Negative very small, Negative small	0.36 0.64 0 0 0
25	1	Negative very small, Zero	1 0 0 0 0
26	1	Negative very small, Positive small	1 0 0 0 0
27	1	Negative very small, Positive medium	1 0 0 0 0
28	1	Negative very small, Positive large	1 0 0 0 0
29	1	Zero, Negative large	1 0 0 0 0
30	1	Zero, Negative medium	1 0 0 0 0
31	1	Zero, Negative small	1 0 0 0 0
32	1	Zero, Zero	1 0 0 0 0
33	1	Zero, Positive small	1 0 0 0 0
34	1	Zero, Positive medium	1 0 0 0 0
35	1	Zero, Positive large	1 0 0 0 0
36	1	Positive small, Negative large	0.39 0.61 0 0 0
37	1	Positive small, Negative medium	0.9 0.1 0 0 0
38	1	Positive small, Negative small	1 0 0 0 0
39	1	Positive small, Zero	1 0 0 0 0
40	1	Positive small, Positive small	1 0 0 0 0
41	1	Positive small, Positive medium	1 0 0 0 0
42	1	Positive small, Positive large	1 0 0 0 0
43	1	Positive medium, Negative large	0.1 0.9 0 0 0
44	1	Positive medium, Negative medium	0.3 0.7 0 0 0
45	1	Positive medium, Negative small	0.85 0.15 0 0 0
46	1	Positive medium, Zero	0.98 0.02 0 0 0
47	1	Positive medium, Positive small	1 0 0 0 0
48	1	Positive medium, Positive medium	1 0 0 0 0
49			1 0 0 0 0
50	1	Positive large, Negative large	0.9 0.1 0 0 0
51	1	Positive large, Negative medium	0.99 0.01 0 0 0
52	1	Positive large, Negative small	1 0 0 0 0
53	1	Positive large, Zero	1 0 0 0 0
54	1	Positive large, Positive small	1 0 0 0 0
55	1	Positive large, Positive medium	1 0 0 0 0
56	1	Positive large, Positive large	1 0 0 0 0

As described in the above sections, the data set may not cover all patterns about flow difference and pressure difference, which means that perhaps only a part of the rules shown in Table 13 can be activated. To prove this point, the samples of oil pipeline leaks are entered into the above BRB expert system. The samples are divided into training data set and testing data set. The training data set includes 510 samples, which are selected from the 2007 samples regularly, and the remaining 1497 samples are used as a testing data set. P-CMA-ES algorithm is utilized as an optimization tool of the BRB expert system, and to ensure the fairness of the optimization process, the parameters and iterations are the same for different conditions.

#### Case 1-optimization process of BRB using activation rates

**Figure 14:**
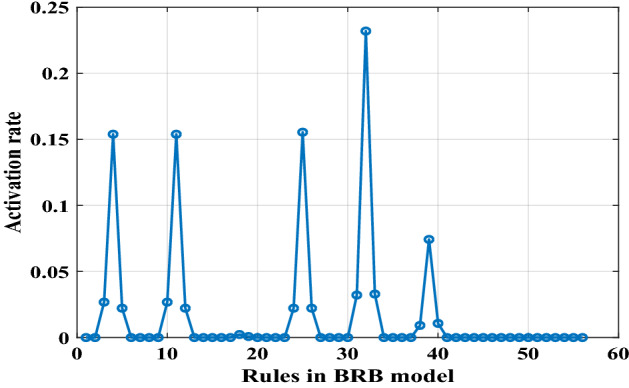
The activation rates of 56 rules in the BRB expert system.

Figure 14 shows the activation rates of 56 rules in the BRB expert system when the training data set are entered. It can be seen that only a few rules are activated and their activation times of them are different.

Four BRBs with different activation thresholds were established to compare with traditional BRBs. In these BRBs, rules with activation rates greater than the threshold are selected to participate in the optimization process. Therefore, different thresholds preserve different numbers of rules. The optimization algorithm is P-CMA-ES with an iteration time of 100. Table 14 shows the details and test results for different BRBs. Where BRB stands for traditional BRB, BRB_1 to BRB_4 represents four BRBs with different activation thresholds, BRB_SQP stands for BRB using Sequential Quadratic Programming optimization algorithm, BRB_PSO stands for BRB using Particle Swarm Optimization algorithm, BRB_S stands for BRB using only the selected rules.

**Table 14:**
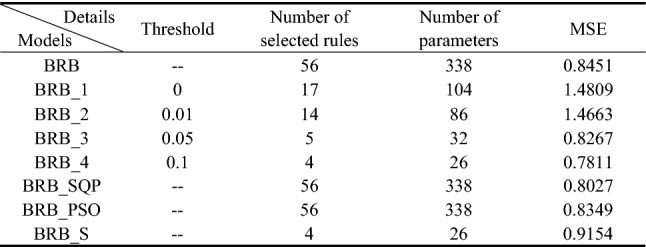
The details and final testing results generated by different BRBs.

It can be seen that BRB_4 with minimum rules generates the best result. The mean square error (MSE) of BRB_4 with 26 optimization parameters is even smaller than traditional BRB with 338 optimization parameters.

**Figure 15:**
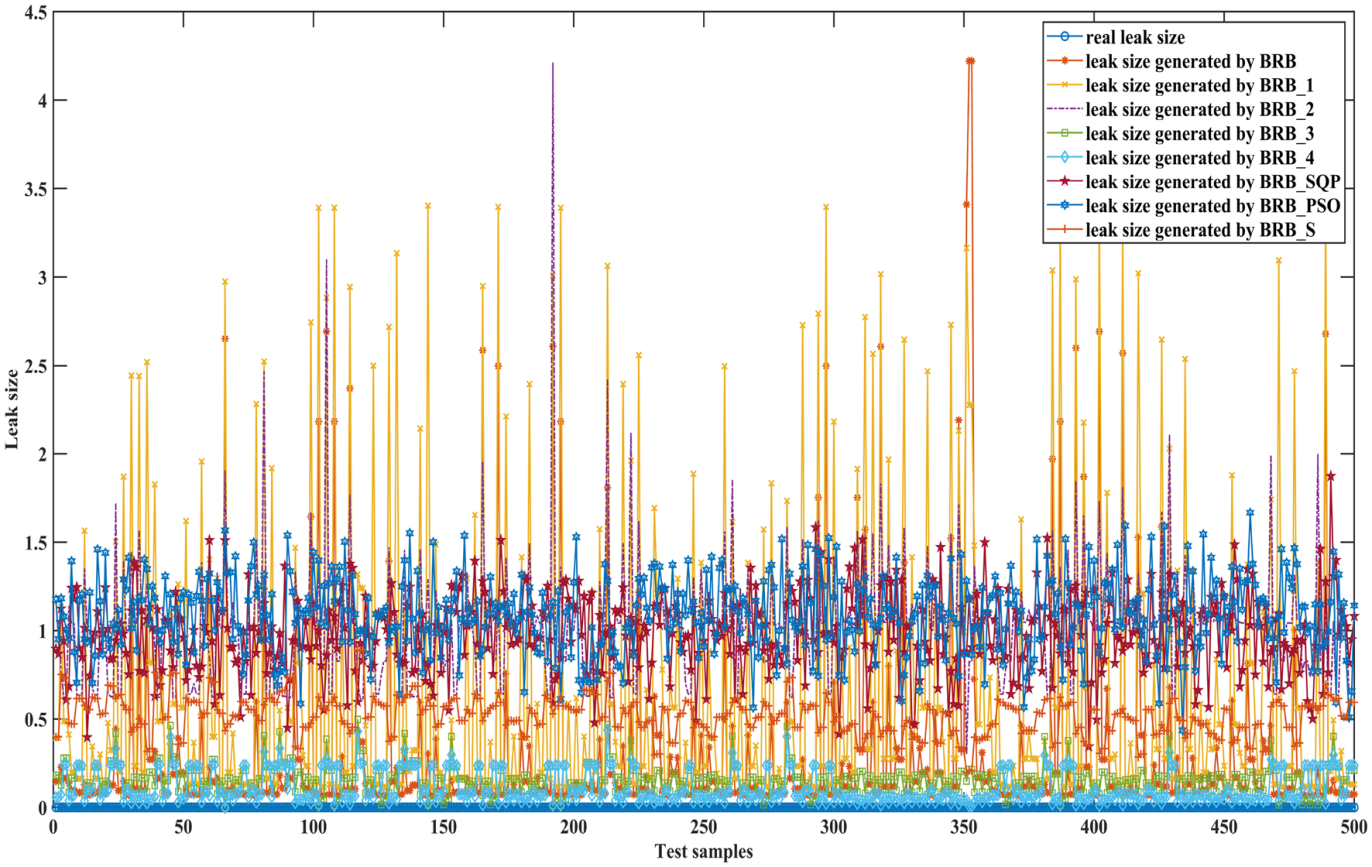
The 1–500 comparative results generated by different BRBs.

**Figure 16:**
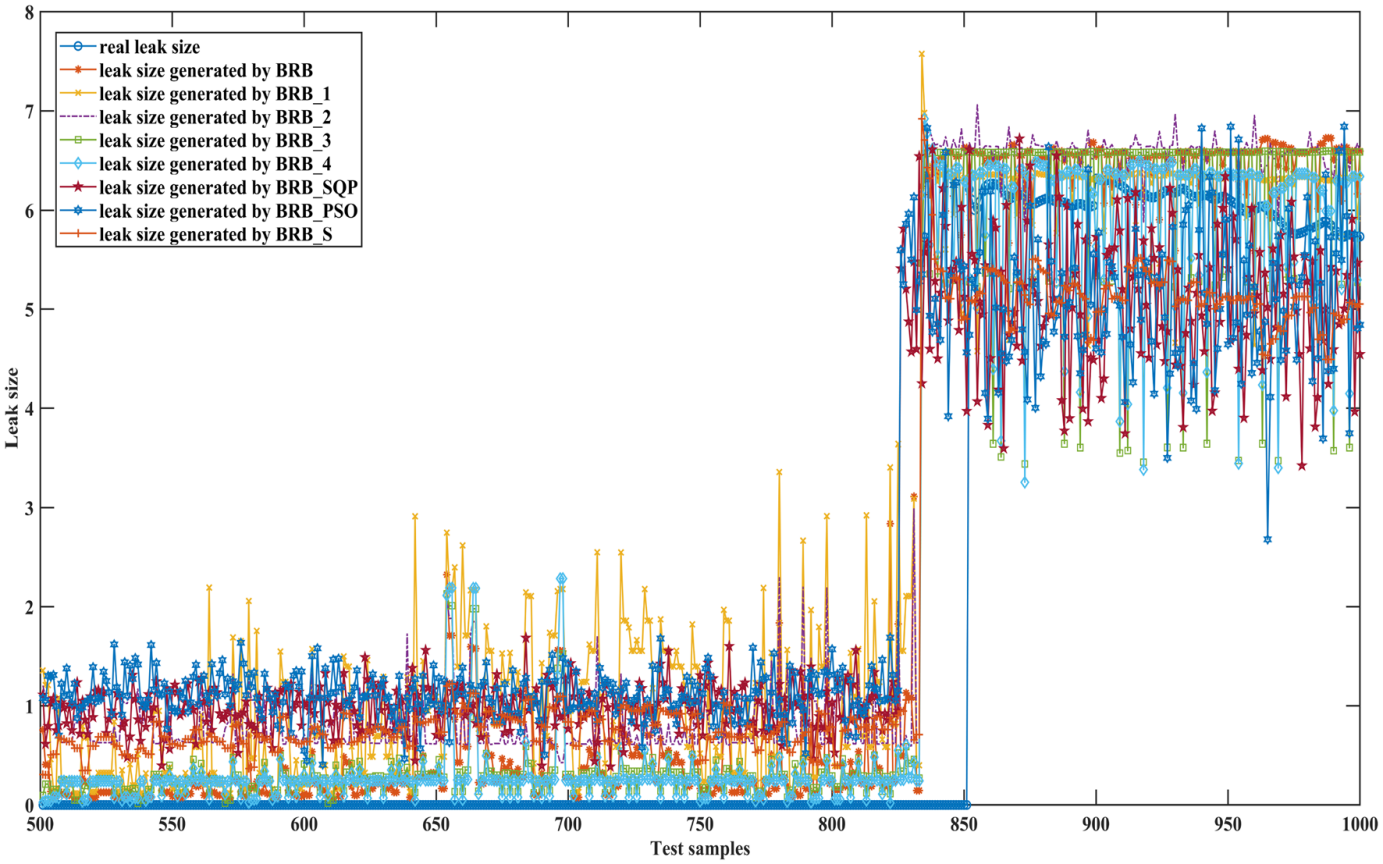
The 501–1000 comparative results generated by different BRBs.

**Figure 17:**
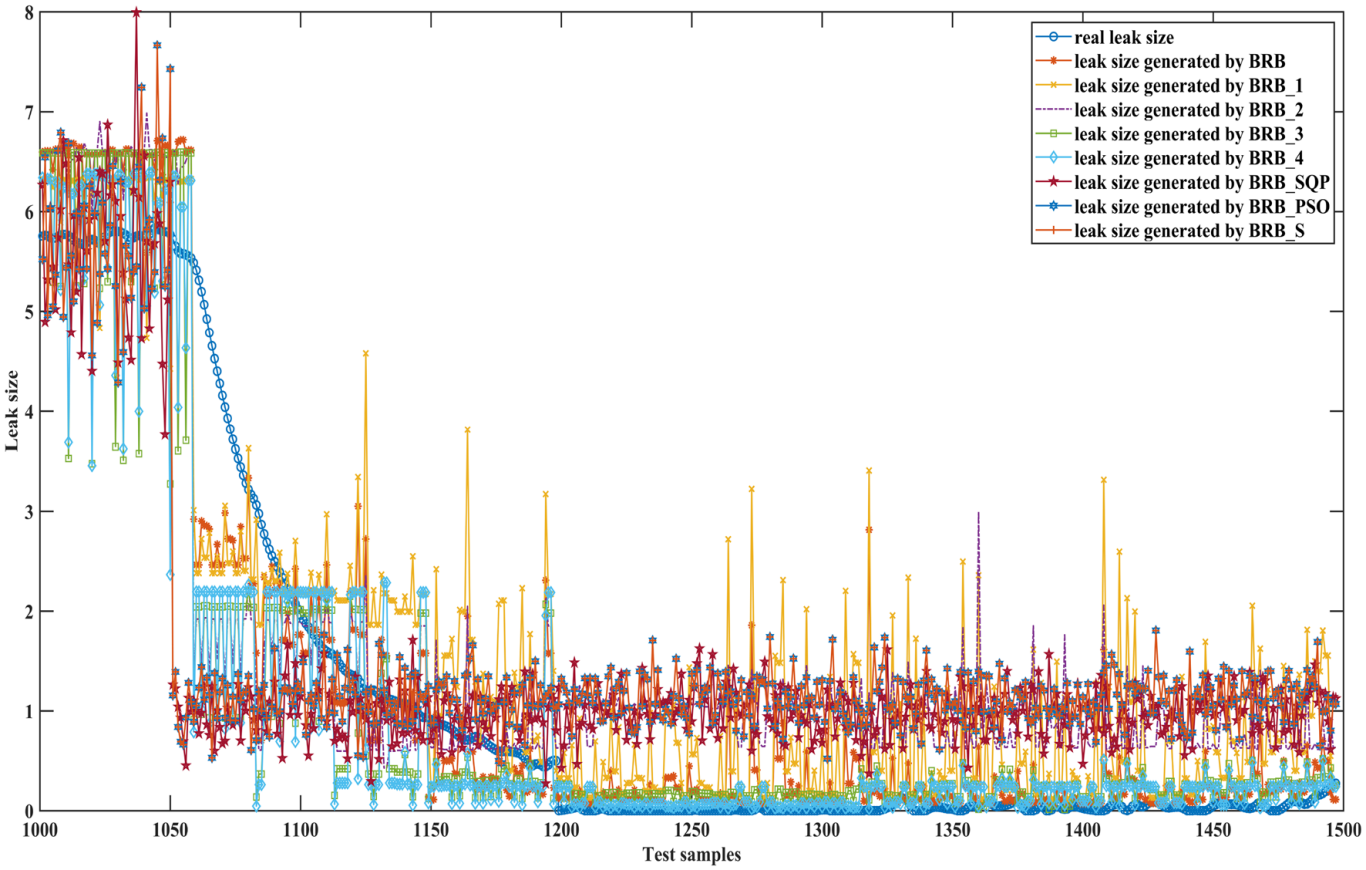
The 1001–1497 comparative results generated by different BRBs.

Figures 15, 16 and 17 can better demonstrate the results of comparative experiments, where 1497 samples are divided into three parts.

#### Case 2-parallel processing for BRB using activation rates

**Figure 18:**
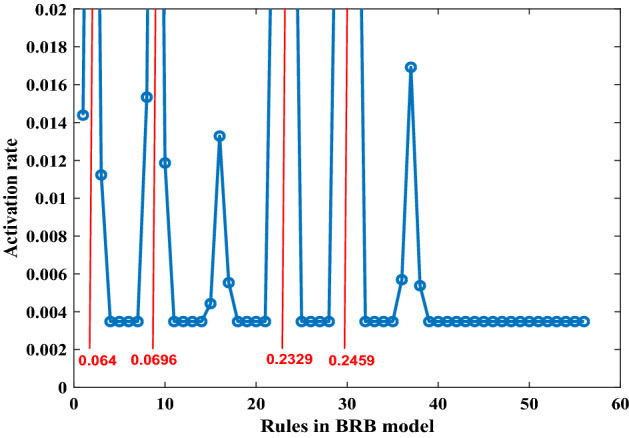
The activation rates of 56 rules generated by all samples.

In this section, all samples are treated as training data to activate more rules in BRB, which is to start up the parallel optimization process of BRB. Figure 18 shows the activation rates generated by training data. It can be seen that the activation rates of 56 rules are more balanced than activation rates shown in Fig. 14 of case 4.2.1. In Fig. 18, most rules have been activated, although some rules are activated less frequently. When more rules participate in the optimization process, the number of parameters to be optimized will increase dramatically. Therefore, the method of the decomposing data set is proposed to generate independent optimization sub-processes.

**Figure 19:**
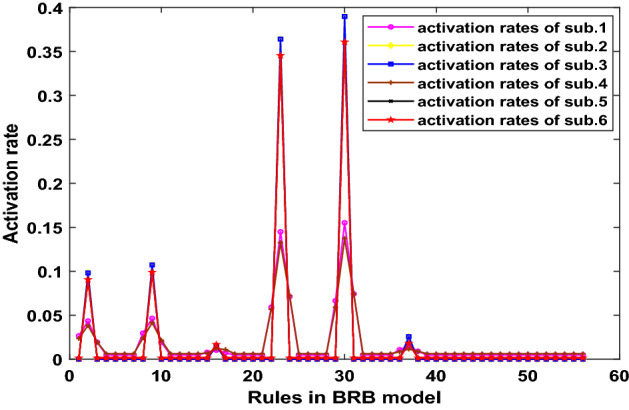
The activation rates of 56 rules generated by sub.1–6.

By using the parallel optimization method described in “Parallel optimization method of BRBs” section, the training data sets are divided into 6 parts, each of which belongs to the corresponding 6 sub-BBR models, denoted as sub.1–6. Thus, the parallel optimization processes can be also divided into 6 parts. The operating environment uses MATLAB parallel toolkit with Intel Core i7-8750H 2.2 GHz CPU, 16 GB memory. The optimization algorithm is P-CMA-ES, and the iteration time is 100. The activation rates of 56 rules generated by sub.1–6 are shown in Fig. 19.

In this case, the activation rate threshold of the parallel optimization is 0.002, then the number of the rules involved in optimization of each sub-BRB are shown in Table 15.

**Table 15:** The number of rules involved in optimization in each sub-BRB.

Sub-BRBs	sub1	sub2	sub3	sub4	sub5	sub6
The number of rules	56	6	6	56	6	6

Thus, the 6 sub-BRBs are assigned to 6 optimization sub-processes and optimized independently. The comparison results between original BRB without using activation rate and optimization parallelization, BRB with activation rate but without optimization parallelization (non-parallelization BRB_a), and BRB with activation rate and optimization parallelization (parallelization BRB_a) are shown in Table 16.

**Table 16:**
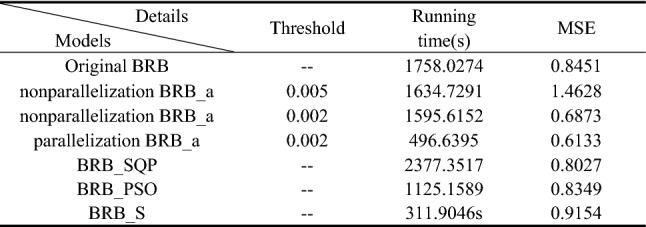
The comparison results between different BRBs.

In Table 16, parallelization BRB_a uses the least time to get the best results. Its running time is about one-fourth of other models. It can be predicted that with the increase of the number of processors, the running time will decrease without affecting the accuracy.

The experimental results show that the BRB expert system with activation rate has higher accuracy than the initial BRB or other BRB optimization algorithms in the process of oil pipeline leakage size assessment, and it takes less time. It effectively solves the problem of BRB expert system combination explosion.

## Conclusion

To solve the combinatorial explosion problem of the BRB expert system, a novel optimization method is proposed in this paper. The proposed method can be applied in two situations: (1) Only a few rules in BRB are activated. In this case, activation rate is used to prune the rules that have never been activated or are inadequately activated. The initial parameters of these removed rules are given by experts, which will not only keeps the role of expert experience but also enhance the credibility of the results. (2) Most rules in BRB are activated. In this case, parallel optimization is proposed by decomposing training data, and activation rate is utilized in each parallel optimization sub-process. The final results of BRB can be obtained by the weighted average method. The proposed optimization method for the BRB expert system can be applied to all kinds of BRB models and can increase the accuracy while reducing the calculation pressure by reducing the parameters and using parallel operation.

## Data Availability

The datasets generated and/or analysed during the current study are not publicly available due Laboratory requirements but are available from the corresponding author on reasonable request.
